# Immunosuppressive Therapy After Autologous Hematopoietic Stem Cell Transplantation in Systemic Sclerosis Patients—High Efficacy of Rituximab

**DOI:** 10.3389/fimmu.2021.817893

**Published:** 2022-01-17

**Authors:** Michael Gernert, Hans-Peter Tony, Matthias Fröhlich, Eva Christina Schwaneck, Marc Schmalzing

**Affiliations:** ^1^ Department of Medicine II, Rheumatology and Clinical Immunology, University Hospital of Würzburg, Würzburg, Germany; ^2^ Rheumatology and Clinical Immunology, Asklepios Klinik Altona, Hamburg, Germany

**Keywords:** systemic sclerosis, scleroderma, autologous hematopoietic stem cell transplantation, immunosuppression, adverse events, rituximab

## Abstract

**Background:**

Systemic sclerosis (SSc) patients often need immunosuppressive medication (IS) for disease control. If SSc is progressive despite IS, autologous hematopoietic stem cell transplantation (aHSCT) is a treatment option for selected SSc patients. aHSCT is effective with good available evidence, but not all patients achieve a treatment-free remission after aHSCT. Thus far, data about the need of IS after aHSCT in SSc is not published. The aim of this study was to investigate the use of IS after aHSCT, its efficacy, and the occurrence of severe adverse events (SAEs).

**Methods:**

Twenty-seven patients with SSc who had undergone aHSCT were included in this single-center retrospective cohort study. Clinical data, including IS, SAEs, and lung function data, were collected.

**Results:**

Sixteen of 27 (59.3%) patients received IS after aHSCT. Methotrexate, rituximab, mycophenolate, cyclophosphamide, and hydroxychloroquine were most commonly used. The main reason for starting IS was SSc progress. Nine patients received rituximab after aHSCT and showed an improvement in modified Rodnan skin score and a stabilization of lung function 2 years after rituximab. SAEs in patients with IS after aHSCT (50.0%) were not more common than in patients without IS (54.6%). SAEs were mostly due to SSc progress, secondary autoimmune diseases, or infections. Two deaths after aHSCT were transplantation related and three during long-term follow-up due to pulmonary arterial hypertension.

**Conclusion:**

Disease progression and secondary autoimmune diseases may necessitate IS after aHSCT in SSc. Rituximab seems to be an efficacious treatment option in this setting. Long-term data on the safety of aHSCT is reassuring.

## Background

The pathogenesis of systemic sclerosis (SSc) comprises fibrosis, inflammation, and vasculopathy (1). These three components of SSc pathogenesis often need to be addressed with different treatment modalities. SSc patients with lung fibrosis can be treated with nintedanib (2). Inflammation can be treated with immunosuppressive medications (IS). The European League against Rheumatism (EULAR) recommendations (3) include methotrexate (MTX), which can improve skin sclerosis in early forms of SSc (4), and cyclophosphamide (CYC), which can stabilize SSc interstitial lung disease (5). Further IS, which showed some efficacy on disease manifestations, are mycophenolate (MMF) (6), rituximab (RTX) (7–9), and tocilizumab (10). Autologous hematopoietic stem cell transplantation (aHSCT) has the best evidence for effective treatment of SSc with the three randomized controlled trials ASSIST (11), ASTIS (12), and SCOT (13). These showed that aHSCT is superior to intravenous cyclophosphamide regarding skin and lung involvement, quality of life, and overall survival. However, not all SSc patients achieve a treatment-free remission of SSc after aHSCT. Progressive disease despite aHSCT confronts the practitioner with great challenges. Thus far, no data or guidelines are published about the need and use of IS after aHSCT in SSc. Therefore, the aim of the present study was to describe the indication for IS and the prevalence of the use of IS after aHSCT in SSc and additionally to record the occurrence of severe adverse events (SAEs).

## Patients and Methods

### Patients and Data Collection

All of the SSc patients who had undergone aHSCT and were followed up at our center were included in this study. The cohort comprised 27 patients who met the American College of Rheumatology (ACR)/EULAR criteria (14) for SSc. Data were collected between the years 2009 and 2021. All immunosuppressive medications initiated after aHSCT were collected, apart from glucocorticoids. SAEs, i.e., necessity for hospitalization or death, were recorded. Data were taken from the patients’ electronic files (EMIL by itc‐ms.de, Marburg, Germany and SAP SE, Walldorf, Germany).

### Myeloablative Autologous Hematopoietic Stem Cell Transplantation

Patients were treated according to the ASTIS trial protocol (12) with modifications as previously described (15, 16): 2 g/m^2^ cyclophosphamide with at least 105 µg granulocyte-colony stimulating factor daily from day 2 after cyclophosphamide was given for mobilization of autologous hematopoietic stem cells. Leukapheresis followed. CD34^+^ selection was done by immunomagnetic separation (CliniMACS CD34 Complete Kit, Miltenyi Biotec, Bergisch Gladbach, Germany). As immunoablative conditioning regimen over days 1–4, a total of 200 mg/kg body weight (bw) cyclophosphamide, and over days 2–5, a total of 30 mg/kg bw rabbit anti-thymocyte globulin (ATG; Grafalon, Neovii Biotech, Gräfeling, Germany) were given. On day 6, at least 2.0 × 10^6^ CD34^+^ autologous hematopoietic stem cells/kg bw were reinfused.

### Statistical Analysis

Calculations were done with SPSS Statistics v 26.0 (IBM, Armonk, NY). Shapiro–Wilk tests were used to test for normal distribution. When normal distribution was absent, medians with interquartile ranges (IQR) were calculated. For continuous variables, differences between paired groups were examined with Wilcoxon signed-rank tests and differences between unpaired groups with Mann–Whitney U-tests. For metrical variables, differences between unpaired groups were calculated with Fisher’s exact tests. Differences were considered significant when two tailed *p-*values were <0.05. Excel (Microsoft, Redmond, Washington) was used to collect the data and draw the graphs. Figures were grouped with Photoshop (Adobe, San Jose, CA).

## Results

### Patients’ Characteristics Before aHSCT and Transplantation Parameters

Twenty-seven SSc patients, who are in our care after aHSCT, were included in the study. Of the patients, 48.1% were female, with a median age of 47.2 years and a median disease duration of 25.0 months before aHSCT; 93.3% were positive for anti-nuclear antibodies (ANA), and 74.1% were positive for Scl-70 antibodies; 88.9% had a diffuse cutaneous form (dcSSc) with a mean modified Rodnan skin score (mRSS) of 23.0; and 37.0% had ever smoked (active smokers during aHSCT were 7.4%). Pulmonary fibrosis on thoracic computed tomography was present in 77.8% of the patients. Cardiac involvement (i.e., high-sensitive troponin above the upper limit of normal + myocardial late enhancement in cardiac MRI or myocarditis in myocardial biopsy) was present in 44.4%; 25.9% had a history of pulmonary arterial hypertension (PAH), which was well controlled at the beginning of mobilization.

The indication for aHSCT was progressive skin involvement in 33.3%, progressive lung involvement in 55.6%, and both manifestations in 11.1%. A CD34^+^ selection of stem cells was done in 96.3%. All patients received anti-thymocyte globulin (ATG) during conditioning (**Table 1**).

**Table 1:** Characteristics of the study population and therapy.

Characteristics		Values
Patients’ characteristics before aHSCT		
Female, *n* (%)		13/27 (48.1)
Age at aHSCT, mean (range), years		47.2 (23–64)
Disease duration before aHSCT, median (range), months		25.0 (5–156)
Diffuse cutaneous form, *n* (%)		24/27 (88.9)
mRSS, mean (range), points		23.0 (5–44)
Anti-nuclear antibody positivity, *n* (%)		26/27 (96.3)
Anti-Scl-70 antibody positivity, *n* (%)		20/27 (74.1)
Anti-Centromere antibody positivity, *n* (%)		0/0 (0.0)
Smoking history:	During aHSCT, *n* (%)	2/27 (7.4)
	Ever, *n* (%)	10/27 (37.0)
Pulmonary fibrosis on thoracic computed tomography, *n* (%)		21/27 (77.8)
Cardiac involvement^§^, *n* (%)		12/27 (44.4)
Pulmonary arterial hypertension, *n* (%)		7/27 (25.9)
Transplantation parameters		
Indication for aHSCT:	Skin, *n* (%)	9/27 (33.3)
	Lung, *n* (%)	15/27 (55.6)
	Skin and lung, *n* (%)	3/27 (11.1)
CD34^+^ selection for stem cell autograft, *n* (%)		26/27 (96.3)
ATG use for conditioning regimen, *n* (%)		27/27 (100.0)
Immunosuppression (glucocorticoids not regarded) after aHSCT		
Patients without IS after aHSCT, *n* (%)		11/27 (40.7)
Follow-up time after aHSCT, median (IQR), months		29.0 (10.0–122.0)
Patients with IS after aHSCT, *n* (%)		16/27 (59.3)
Follow-up time after aHSCT, median (IQR), months		67.0 (39.0–124.5)
Cumulative IS-free time after aHSCT, median (IQR), months		29.5 (9.5–49.3)
Proportion of cumulative IS-free time/follow-up time, median (IQR), %		60.3 (18.8–74.0)
IS used:	MTX, *n* (%)	9/27 (33.3)
	Rituximab, *n* (%)	9/27 (33.3)
	Hydroxychloroquine, *n* (%)	3/27 (11.1)
	MMF, *n* (%)	3/27 (11.1)
	Cyclophosphamide, *n* (%)	3/27 (11.1)
	Colchicine, *n* (%)	2/27 (7.4)
	Cyclosporine A, *n* (%)	1/27 (3.7)
	Azathioprine, *n* (%)	1/27 (3.7)
	Tocilizumab, *n* (%)	1/27 (3.7)
	Anakinra, *n* (%)	1/27 (3.7)

aHSCT, autologous hematopoietic stem cell transplantation; ATG, anti-thymocyte globulin; IS, immunosuppression; IQR, interquartile range; MMF, mycophenolate; mRSS, modified Rodnan skin score; MTX, methotrexate.

^§^That is, high-sensitive troponin above upper limit of normal + myocardial late enhancement in cardiac MRI or myocarditis in myocardial biopsy.

### Need of Immunosuppression After aHSCT in SSc

The immunosuppressive medications (IS; except glucocorticoids), which were started after aHSCT, were recorded. Eleven patients (40.7%) did not need any IS after aHSCT within the median follow-up time of 29.0 (IQR, 10.0–122.0) months. Sixteen of the 27 SSc patients (59.3%) needed IS after aHSCT within the median follow-up time of 67.0 (39.0–124.5) months (comparison between median follow-up time between no IS and IS: *p* = 0.148). The 16 patients receiving IS had a median cumulative immunosuppression-free time after aHSCT of 29.5 (9.5–49.3) months. The ratio of immunosuppression-free time to the overall follow-up time resulted in a median of 60.3 (18.8–74.0) %.

Different immunosuppressive drugs were used in the 16 SSc patients, who needed IS after aHSCT: methotrexate (MTX) in nine patients, rituximab (RTX) in nine, hydroxychloroquine (HQC) in three, mycophenolate (MMF) in three, cyclophosphamide (CYC) in three, colchicine in two, and cyclosporine A (CSA), azathioprine (AZA), tocilizumab, and anakinra in one patient each (for summary, see **Table 1**; for details, see **Table 2**). The median time after aHSCT when IS was started was 9.5 (5.3–19.5) months. The most common indication for starting an IS was cutaneous or pulmonary SSc progress in 12 patients. Indications for the start of rituximab were cutaneous progress of SSc (n = 4), pulmonary progress (n = 2), and secondary autoimmune diseases like microscopic polyangiitis (MPA; n = 1), immune thrombocytopenia (n = 1), and myositis (n = 1) (**Table 2**).

**Table 2:** Immunosuppression (IS; without glucocorticoids) and severe adverse events (SAEs) after aHSCT.

Patient	IS before aHSCT	IS after aHSCT, drug, dose, begin (months after aHSCT), intake duration (months), indication	Time without IS after aHSCT, months	Follow-up after aHSCT, months	SAE^#^ Begin after aHSCT (months), treatment	Deathtime after aHSCT (months)
1	CYC, Imatinib	MTX, 15 mg s.c./p.o., 35 mo, 55 mo, cutaneous progress	86	141	Esophageal stenosis, 15 mo, endoscopic dilatation	na
		RTX, 2× 1 g, 44 mo, na, microscopic polyangiitis			Microscopic polyangiitis, 44 mo, RTX	
2	MTX, CSA, MMF, CYC	MTX, 15 mg s.c., 15 mo, 2 mo, cutaneous progress	32	72	Esophageal stenosis, 29 mo, endoscopic dilatation	na
		MMF, 1–3 g, 17 mo, 38 mo, cutaneous progress			Pneumonia, 53 mo, piperacillin/tazobactam	
		RTX, 2× 1g, 23 + 30 + 48 mo, na, cutaneous progress				
		CYC, 1× 750 mg/m^2^, 48 mo, na, pulmonary progress				
3	CYC	RTX, 2× 1 g, 21 + 27 mo, na, pulmonary progress	41	47	None	na
4	MTX, HCQ, CSA	RTX, 4× 375 mg/m^2^, 18 mo, na, immune thrombocytopenia	84	141	Immune thrombocytopenia, 16mo, RTX + CSA	na
		CSA, 150 mg, 20 mo, 50 mo, immune thrombocytopenia			Primary hyperthyroidism, 18 mo, thiamazole	
5	CYC, AZA	MTX, 15 mg s.c., 5 mo, 2 mo, cutaneous progress	29	39	Pneumocystis pneumonia, 18 mo, cotrimoxazole	na
		Tocilizumab, 162 mg s.c., 6 mo, 1 mo, cutaneous progress			Candida esophagitis, 19 mo, fluconazole	
		RTX, 2× 1 g, 6 mo + 14 mo, na, cutaneous progress				
6	MMF	RTX, 2× 1 g, 11 mo, na, cutaneous progress	14	17	None	na
		MTX, 15 mg s.c., 12mo, 3mo, cutaneous progress				
7	MTX, AZA, CYC	RTX, 2x 1g, 2 + 8+16+23+29mo, pulmonary progress	8	39	None	na
8	HCQ, AZA, CYC, RTX	MMF, 2 g, 22 mo, 83 mo, myositis	22	120	Infected finger ulcer, 26 mo, amoxicillin/clavulanic acid	Yes, 120 mo
		RTX, 2× 1 g, 24 + 20 + 99 mo, na, myositis and pulm. Progress			Intestinal bleeding, 90 mo, endoscopy	
					Worsening of PAH, 118 mo, uk	
9	MTX, CSA, MMF	HCQ, 400 mg, 6 mo, 30 mo, cutaneous progress	44	72	None	na
		MTX, 15 mg p.o., 10 mo, 2 mo, cutaneous progress				
		RTX, 2× 1 g, 12 mo, na, cutaneous progress				
		CYC, 1× 750 mg/m^2^, 12 mo, na, cutaneous progress				
10	CYC, MMF	MTX, 15 mgs.c./p.o., 6 mo, 35 mo, inflammatory bursitis	0	41	None	na
11	MTX, CYC	MTX, 15 mg p.o., 8 mo, 6 mo, cutaneous progress	57	64	Desmoid tumor 3rd left rib, 27 mo, excision	na
12	MTX, HQC, CYC	MTX, 10 mg s.c., 2 mo, 5 mo, cutaneous progress	26	36	None	na
13	MTX, AZA, CSA, RTX, leflunomide, MMF, CYC, tocilizumab	HCQ, 400 mg, 3 mo, 2 mo, arthritis	2	31	Pneumonia, 8 mo, moxifloxacin	Yes, 41 mo
		Colchicine, 0.5 mg, 4 mo, 27 mo, CPPD disease			Worsening of PAH, 23 mo, selexipag + iloprost	
		Anakinra, 100 mg, 6 mo, 2 mo, arthritis				
14	CYC, RTX	CYC, 1× 750 mg/m^2^, 18 + 19 + 20 + 21 mo, na, pulmonary progress	30	150	None	na
		MMF, 1–3 g, 23 mo, 108 mo, pulmonary progress				
		Colchicin, 0.5 mg, 99mo, 6mo, arthritis urica				
15	CYC, MMF	MTX, 10 mg p.o., 7 mo, 21 mo, cutaneous progress	51	70	None	na
16	CYC, MMF	AZA, 150 mg, 20 mo, 100 mo, Sjögren’s syndrome	8	126	Sjögren’s syndrome, 8 mo, AZA	na
		HCQ, 400 mg, 30 mo, 96 mo, Sjögren’s syndrome				
17	Apremilast, MTX, CYC	None	10	10	Pneumonia with lactate acidosis, 9 mo, piperacillin/tazobactam	Yes, 10 mo
18	CYC	None	1	1	Pulmonary progress, 1 mo, invasive ventilation	Yes, 1 mo
					Stroke (right A. cerebri media), 1mo, BSC	
19	CYC	None	142	142	None	na
20	HCQ, MMF, RTX	None	18	18	None	na
21	CYC, RTX	None	122	122	Primary hyperthyroidism, 19 mo, thyroidectomy + thiamazole	na
22	CYC	None	39	39	None	na
23	MMF	None	19	19	None	na
24	CYC	None	119	119	Deep vein thrombosis, 4 mo, phenprocoumon	na
25	Sulfasalazine, MTX	None	116	140	Pneumonia, 90 mo, moxifloxacin	
					Lung adenocarcinoma, 116 mo, pembrolizumab + pemetrexed	
26	CYC, CSA, MTX	None	2	2	None	na
27	CYC, RTX, tocilizumab	None	29	29	PAH, 11 mo, sildenafil + riociguat + macitentan + selexipag	Yes, 29 mo
					Pneumogenic sepsis, 14 mo, piperacillin/tazobactam + meropenem	

aHSCT, autologous hematopoietic stem cell transplantation; BSC, best supportive care; CSA, ciclosporine A; CPPD, calcium pyrophosphate crystal deposition; CYC, cyclophosphamide; HCQ, hydroxychloroquine; IS, immunosuppression; MMF, mycophenolate; mo, months; MTX, methotrexate; na not, applicable; PAH, pulmonary arterial hypertension, p.o., per os; RTX, rituximab; s.c., subcutaneous; uk, unknown.

^#^SSc progress is listed in the “IS after aHSCT” column and not in the “SAE” column.

### Prevalence of Severe Adverse Events Does Not Differ Between Patients Taking IS and Patients Without IS

Of the 16 patients who took IS after aHSCT, eight (50.0%) developed a severe adverse event (SAE), which caused hospitalization. The median time after aHSCT, when the first SAE occurred, was 17.0 (9.8–26.8) months. Of the 11 SSc patients not taking IS after aHSCT, six (54.6%) had an SAE at a median of 10.0 (3.3–36.8) months after aHSCT. Neither the prevalence of SAEs was significantly different between the groups (*p* = 1.000) nor the time of occurrence after aHSCT (*p* = 0.414). Relevant reasons for SAEs were SSc progress, autoimmune diseases, infections, and malignancy (**Table 2**). Five autoimmune diseases were recorded: MPA, immune thrombocytopenia, primary hyperthyroidism (two times), and Sjögren’s syndrome. Seven severe infections (six pneumonias and one infected finger ulcer) led to hospitalization. One patient developed malignancy (lung adenocarcinoma) 116 months after aHSCT. Death occurred in five of our patients, of which two were aHSCT-associated (one in the first month after aHSCT due to respiratory insufficiency assumedly because of progressive lung disease and another 10 months after aHSCT due to pneumonia with lactate acidosis), and three were due to respiratory insufficiency because of progressive PAH (29, 41, and 120 months after aHSCT, respectively).

### IS Improves Skin and Stabilizes Lung Function in SSc Patients After aHSCT

**Figure 1:**
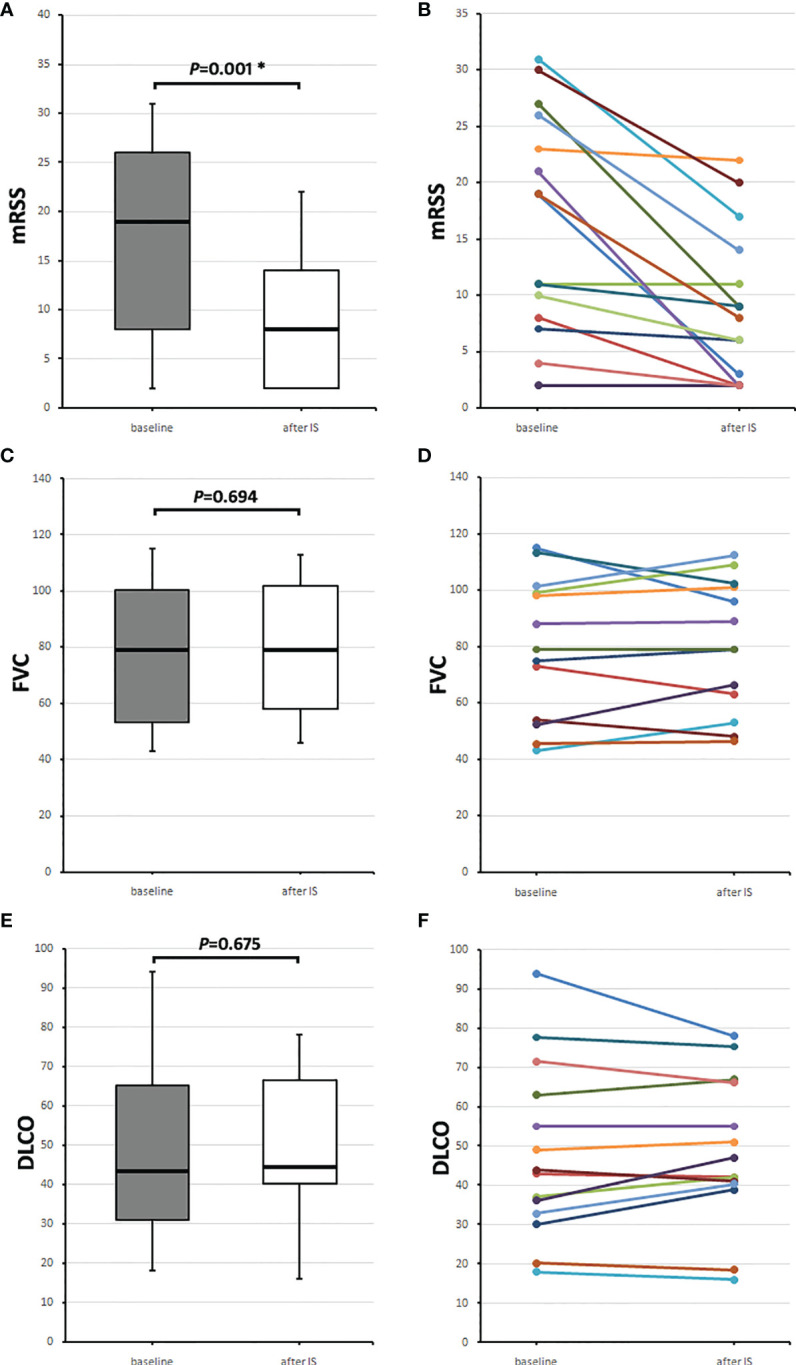
Course of skin and lung parameters of the 16 SSc patients receiving any immunosuppressive medication (IS) after aHSCT. Baseline values (gray boxes) were taken when IS was initiated and follow-up values (after IS, white boxes) 2 years later. **(A)** Median modified Rodnan skin score (mRSS) values and **(B)** individual mRSS values, **(C)** median forced vital capacity (FVC) in percentages of predicted, and **(D)** individual FVC values, and **(E)** median diffusion capacity for carbon monoxide (DLCO) in percentage of predicted with **(F)** individual DLCO values. Box plots show medians with interquartile range, whiskers indicate minimums and maximums; *significant (*p* < 0.05) difference compared to baseline in a Wilcoxon signed-rank test.

The 16 patients, who received IS after aHSCT, were analyzed according to their course of mRSS (n = 15), forced vital capacity (FVC; in percentages of predicted), and diffusion capacity for carbon monoxide (DLCO; in percentage of predicted) (n = 14). Baseline mRSS values were collected at the time when the respective IS was initiated (0.0 months [IQR 0.0–0.0]), and the follow-up value was collected 24.0 (22.0–26.0) months later. Median mRSS was 19.0 (8.0–29.0) points at baseline and 8.0 (2.0–14.0) points, 2 years after initiation of IS (*p* = 0.001) (**Figure 1A**).

Baseline FVC and DLCO were collected at the time of IS initiation (0.0 [−2.0–0.0] months) and 23.0 (22.0–28.0) months after starting IS. Median baseline FVC was 79.0 (53.1–100.3) % and 2 years later 79.0 (58.0–101.7) % (*p* = 0.694) (**Figure 1B**). Baseline DLCO was 43.5 (31.1–65.2) % and 2 years later, 44.5 (40.1–66.4) % (*p* = 0.675) (**Figure 1C**).

**Figures 1A, C, E** show medians with IQRs of the patients, who received IS after aHSCT, and **Figures 1B, D, F**, the values for each patient.

In contrast, SSc patients who did not receive IS after aHSCT (n = 7) exhibited no improvement of mRSS (12.0 [12.0–40.0] months after aHSCT the mRSS was 6.0 [4.0–17.0]; 28.0 [23.0–119.0] months after aHSCT the mRSS was 7.0 [4.0–14.0], *p* = 0.450). For details see, **Supplementary Figure S1**.

### SSc Patients Receiving Rituximab After aHSCT Show Improved Skin and Stabilized Lung Function

Nine (of the 16 patients receiving any IS) received rituximab after aHSCT and underwent subgroup analysis according to their course of mRSS, FVC, and DLCO. The median time after aHSCT, when RTX was given for the first time, was 18.0 (8.5–23.5) months.

**Figure 2:**
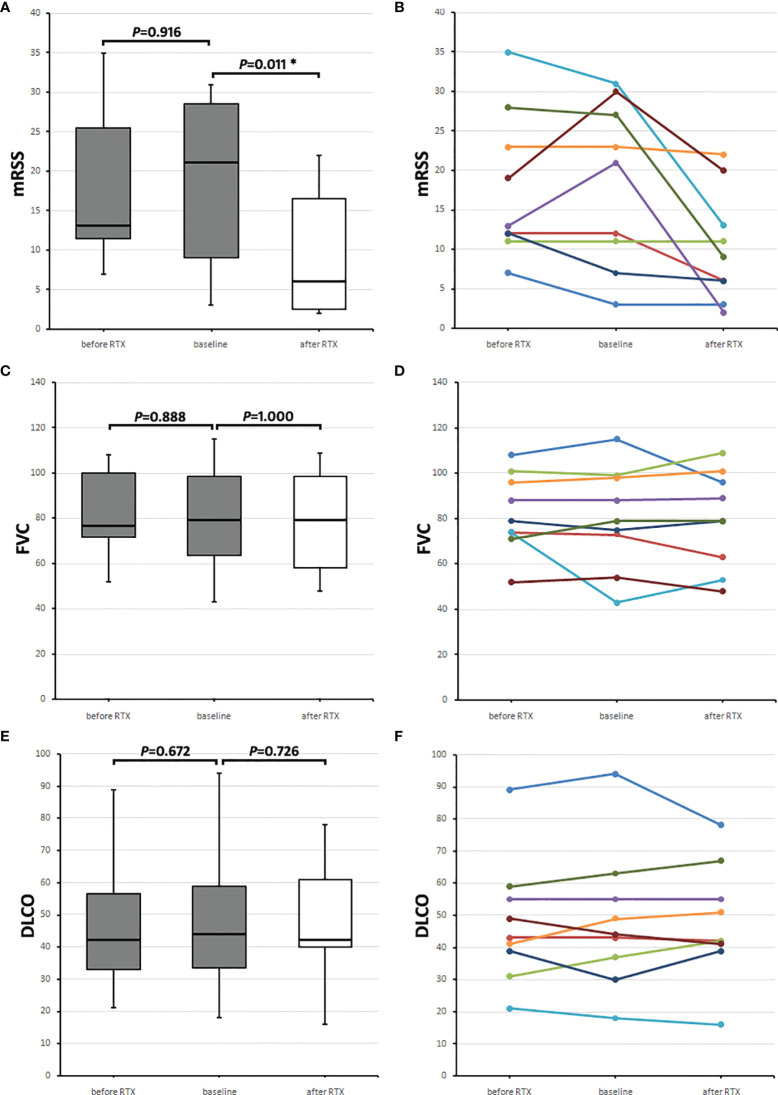
Course of skin and lung parameters of the nine SSc patients receiving rituximab (RTX) after aHSCT. Values before RTX (before RTX, gray boxes) are shown, and baseline values (gray boxes) were taken when RTX was initiated and follow-up values (after RTX, white boxes) 2 years later. **(A)** Median modified Rodnan skin score (mRSS) and **(B)** mRSS values of each of the nine RTX receivers. **(C)** Median forced vital capacity (FVC) in percentages of predicted and **(D)** individual FVC values of each patient. **(E)** Median diffusion capacity for carbon monoxide (DLCO) in percentage of predicted and **(F)** individual DLCO values of each patient. Box plots show medians with interquartile range, whiskers indicate minimums and maximums; *Significant (*p* < 0.05) difference compared to baseline in a Wilcoxon signed-rank test.

mRSS values were collected before RTX application (−5.0 [−1.5 to −11.0] months), at the time of RTX application (baseline; 0.0 [−0.5 to 0.5] months), and after RTX (24.0 [18.5–26.0] months). The mRSS before RTX was 13.0 (11.5–25.5), and baseline mRSS was 21.0 (9.0–28.5) points, and 2 years after, RTX median mRSS was 6.0 (2.5–16.5) points (mRSS before RTX vs. baseline *p* = 0.916, baseline vs. after RTX *p* = 0.011) (**Figures 2A, B**).

FVC (in percentage of predicted) values were collected before RTX application (−9.0 [−10.8 to −4.3] months, at the time of RTX application (baseline; 0.0 [−3.5 to 0.8] months) and 2 years after RTX (24.5 [14.5–29.5] months). The median FVC before RTX of the nine SSc patients was 76.5 (71.8–99.8) %, the baseline FVC was 79.0 (63.5–98.5) %, and 2 years after RTX, it was 79.0 (58.0–98.5) % (FVC before RTX vs. baseline *p* = 0.888, baseline vs. after RTX *p* = 1.000) (**Figures 2C, D**).

DLCO (in percentage of predicted) values were collected before RTX application (−9.0 [−10.8 to −4.3] months), at the time of RTX application (baseline; 0.0 [−3.5 to 0.8] months), and 2 years after RTX (26.0 [14.5 to 29.5] months). Median DLCO before RTX was 42.0 (33–56.5) %, baseline DLCO was 44.0 (33.5–59.0) %, and 2 years after RTX, it was 42.0 (40.0–61.0) % (DLCO before RTX vs. baseline *p* = 0.672, baseline vs. after RTX *p* = 0.726) (**Figures 2E, F**). **Figures 2A, C, E** show medians with IQRs of the nine patients and **Figures 2B, D, F** the values for each patient.

### Use of Rituximab After aHSCT in SSc Patients for Non-skin and Non-lung Indications Is Effective

One SSc patient received rituximab because of the development of a microscopic polyangiitis (MPA) starting 44 months after aHSCT with positivity for myeloperoxidase anti-neutrophil cytoplasmic antibodies (MPO-ANCA) and with renal involvement proven by renal biopsy [for details, see the formerly published case report (17)]. At the time of rituximab application, hematuria and proteinuria were present and improved 26 months after rituximab (erythrocytes/µl urine, 39 vs. 10 [reference, <25]; total protein mg/g creatinine in urine, 524 vs. 102 [reference, <70]; albumin mg/g creatinine in urine, 471 vs. 50 [reference, <30]). A decline was observed in the MPO-ANCA titer (IU/ml, 43 vs. 9 [reference, <3.5]) (**Supplementary Figure S2A**). Another patient received rituximab because of the development of an immune thrombocytopenia 18 months after aHSCT. The thrombocytes were 28,000/µl (reference, 150,000–450,000/µl) at the time of rituximab application and increased to 142,000/µl 24 months after rituximab (**Supplementary Figure S2B**). A third patient received rituximab because of the development of myositis (the diagnosis based on creatine kinase (CK) elevation, proximal muscle weakness, and pathological electromyography) 22 months after aHSCT. The CK value was 467 U/L [reference, <190] at the time of rituximab application and 198 U/L 34 months after rituximab (**Supplementary Figure S2C**).

## Discussion

This study is the first description of the use and efficacy of immunosuppressive medication after aHSCT in SSc patients. We found a high prevalence of 59.3% among our SSc patients, who needed IS after aHSCT, mostly due to SSc progress. An increase in SAEs compared to SSc patients without use of IS could not be found. The IS receivers had improved skin and stabilized lung parameters 2 years after initiation of IS. As most of the patients received more than one IS in the course of time, attributing these effects to one specific IS difficult. However, subgroup analysis of the rituximab receivers (nine of our 16 patients) also showed improved skin and stabilized lung parameters 2 years after rituximab application.

Although the indications for RTX treatment were diverse and were not only due to progressive skin involvement, all of the RTX receivers showed an improvement of mRSS. It cannot be excluded that this improvement was promoted by a positive long-term effect of aHSCT but before RTX application the mRSS was stable (or progressively worsened due to progressive skin involvement).

The prevalence of IS use after aHSCT in our SSc cohort might have been underestimated, as the follow-up time in the group that received IS was considerably longer (67 months) than in the IS-free group (27 months), although this finding was statistically not significant. The IS-free patients might need IS in a longer follow-up period. Our data described that aHSCT cannot achieve a treatment-free remission in all SSc patients.

The aHSCT aims to achieve a reset of the immune system (18) and thereby promote its positive effects on SSc disease manifestations. The reset has been described within the B-cell compartment, as aHSCT induces a decrease in memory B cells and an increase in naive B cells (15). These changes were present for at least 1 year after aHSCT. The reset of the immune system seems not to last in all patients in the long term. A recurrence or persistence of autoreactive lymphocytes has to be assumed (19). This could be an explanation why SSc patients exhibit disease progress after aHSCT and need immunosuppression. To date, no data are available on which treatment option is the best after aHSCT; often treatment strategies are used that are recommend for not-transplanted SSc patients. We found that rituximab had positive effects on skin and lung manifestations of SSc. Rituximab causes similar changes in B-cell subsets as aHSCT with a reduction in memory B cells and increased naive B cells (20, 21). Therefore, rituximab application after aHSCT is a reasonable treatment option for progressive disease, and it is not surprising that our patients benefited from rituximab application.

Long-term follow-up after aHSCT in SSc is described, and event-free survival was described in 64% of the patients after 5 years (22). SSc progress or relapses were described in 28% of the initial responders within 2.7 years, and corticosteroids, mycophenolate, and cyclophosphamide were used for treatment. In the SCOT trial, the initiation of disease-modifying anti-rheumatic drugs was reported in 9% of SSc patients 2 years after aHSCT (13). In other autoimmune diseases, also scarce data are available for the need and use of IS after aHSCT. In multiple sclerosis in a study of 10 patients, two needed IS after aHSCT within the follow-up time of 10 years (23).

Our study is limited due to the small sample size and the retrospective and the single-center study design.

## Conclusion

aHSCT seems to cause a long-lasting disease control in a subgroup of SSc patients. Another subgroup needs temporary immunosuppressive treatment despite aHSCT. Close and long-lasting follow-up for SSc patients seems to be necessary to detect progressive disease early and to treat it appropriately. Further studies evaluating the efficacy of rituximab after aHSCT should be performed.

## Data Availability Statement

The raw data supporting the conclusions of this article will be made available by the authors, without undue reservation.

## Author Contributions

MG had full access to all of the data in the study and takes responsibility for the integrity of the data and the accuracy of the data analysis. Study conception and design: MG and MS. Acquisition of data: MG, MS, and MF. Analysis and interpretation of data: MG, MS, H-PT, and ES. All authors contributed to the article and approved the submitted version.

## Funding

This publication was supported by the Open Access Publication Fund of the University of Würzburg.

## Conflict of Interest

MG received travel grants from AbbVie, Chugai, Eli Lilly, Hexal, Janssen-Cilag, Novartis, Pfizer, and compensation for board memberships from Takeda. H-PT received speaker’s fees, travel grants, research funding, or compensation for consultancies or board memberships from AbbVie, BMS, Chugai/Roche, Eli Lilly, Gilead, Janssen, Novartis, Sandoz/Hexal, Sanofi Aventis, UCB, and Takeda (Shire). ES received speaker’s fees, travel grants, research funding, or compensation for consultancies or board memberships from AbbVie, Chugai/Roche, Janssen-Cilag, Eli Lilly, Novartis, Pfizer, and Takeda (Shire). MF received travel grants from AbbVie, Novartis, Janssen, Eli Lilly, and compensation for board memberships from AbbVie. MS received speaker’s fees, travel grants, research funding, or compensation for consultancies or board memberships from AbbVie, Actelion, Amgen, AstraZeneca, BMS, Boehringer/Ingelheim, Celgene, Chugai/Roche, Eli Lilly, EUSA-Pharma, Genzyme, Gilead, Hexal/Sandoz, Janssen-Cilag, medac, MSD, Mylan, Novartis, Pfizer, Sanofi Pasteur, Takeda (Shire), and UCB.

## Publisher’s Note

All claims expressed in this article are solely those of the authors and do not necessarily represent those of their affiliated organizations, or those of the publisher, the editors and the reviewers. Any product that may be evaluated in this article, or claim that may be made by its manufacturer, is not guaranteed or endorsed by the publisher.

## Supplementary Material

Supplementary Figure 1 Course of skin and lung parameters of 7 SSc patients, who did not receive immunosuppression after aHSCT. The mRSS did not change after aHSCT (12.0 [12.0-40.0] months after aHSCT the median mRSS was 6.0 [4.0-17.0]; 28.0 [23.0-119.0] months after aHSCT the mRSS was 7.0 [4.0-14.0], *P* = 0.450). The median FVC and DLCO (each in percentage of predicted) did not show significant differences 14.0 (12.0-40.0) months after aHSCT compared to 29.0 (23.0-119.0) months after aHSCT (FVC: 65.0 [52.0-87.0] % vs 76.0 [66.0-87.0] %, *P* = 0.674; DLCO: 30.0 [27.0-51.0] % vs 34.0 [23.0-48.0] %, *P* = 0.865). The individual data is shown for **(A)** mRSS, **(B)** FVC, and **(C)** DLCO. The cohort of SSc patients after aHSCT, who did not receive IS, comprised 11 patients, but retrospective data was not available from 4 patients due to short follow up or because of fatal complications in the course of disease.

Supplementary Figure 2 Improvements of non-skin and non-lung parameters in SSc patients with former aHSCT after rituximab (RTX) application. **(A)** Course of urine parameters and of MPO-ANCAs of one SSc patient, who had developed a microscopic polyangiitis after aHSCT and therefore received RTX. **(B)** Course of thrombocytes of one SSc patient, who developed immune thrombocytemia and therefore received RTX. **(C)** Course of creatine kinase (CK) of a SSc patient, who developed myositis after aHSCT and therefore received RTX; mo, months; MPO-ANCA, myeloperoxidase anti-neutrophilic cytoplasmic antibodies.

## Abbreviations

ACR, American College of Rheumatology; aHSCT, autologous hematopoietic stem cell transplantation; ASSIST, American Scleroderma Stem Cell Versus Immune Suppression Trial; ASTIS, Autologous Stem Cell Transplantation International Scleroderma Trial; ATG, anti-thymocyte globulin; bw, body weight; CT, computed tomography; dcSSc, diffuse cutaneous form of systemic sclerosis; EULAR, The European League Against Rheumatism; IQR, interquartile range; mRSS, modified Rodnan skin score; na, not applicable; SCOT, Scleroderma: Cyclophosphamide or Transplantation trial; SSc, systemic sclerosis.
