# Supplementary figures and images for: Immunosuppressive Therapy After Autologous Hematopoietic Stem Cell Transplantation in Systemic Sclerosis Patients—High Efficacy of Rituximab
Source: Front Immunol. 2022 Jan 17;12:817893. doi: 10.3389/fimmu.2021.817893 (PMC8801940; doi:10.3389/fimmu.2021.817893)

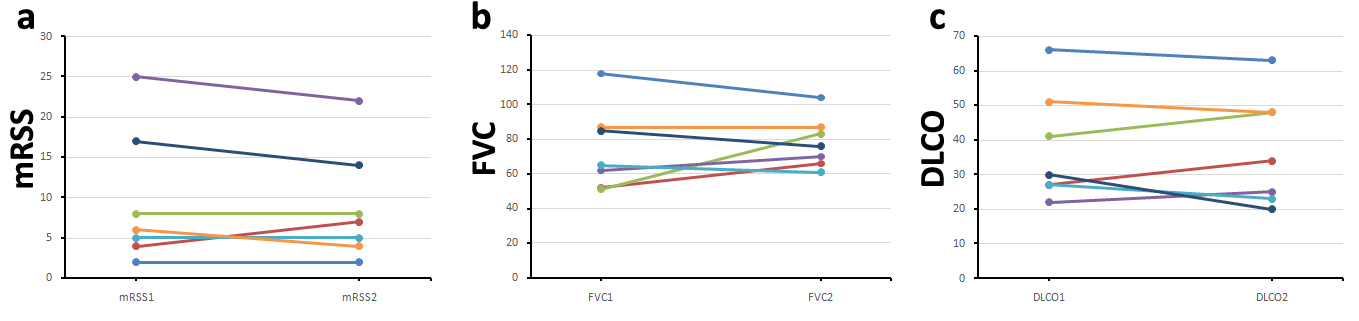

Supplement: Supplementary Figure 1 — Course of skin and lung parameters of 7 SSc patients, who did not receive immunosuppression after aHSCT. The mRSS did not change after aHSCT (12.0 [12.0-40.0] months after aHSCT the median mRSS was 6.0 [4.0-17.0]; 28.0 [23.0-119.0] months after aHSCT the mRSS was 7.0 [4.0-14.0], P = 0.450). The median FVC and DLCO (each in percentage of predicted) did not show significant differences 14.0 (12.0-40.0) months after aHSCT compared to 29.0 (23.0-119.0) months after aHSCT (FVC: 65.0 [52.0-87.0] % vs 76.0 [66.0-87.0] %, P = 0.674; DLCO: 30.0 [27.0-51.0] % vs 34.0 [23.0-48.0] %, P = 0.865). The individual data is shown for (A) mRSS, (B) FVC, and (C) DLCO. The cohort of SSc patients after aHSCT, who did not receive IS, comprised 11 patients, but retrospective data was not available from 4 patients due to short follow up or because of fatal complications in the course of disease. [file Image_1.tif]

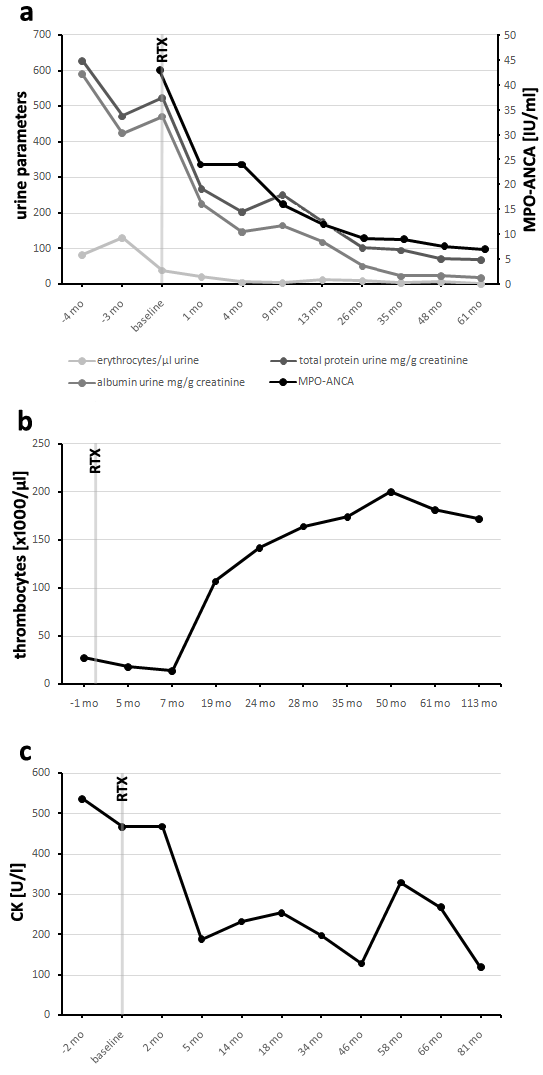

Supplement: Supplementary Figure 2 — Improvements of non-skin and non-lung parameters in SSc patients with former aHSCT after rituximab (RTX) application. (A) Course of urine parameters and of MPO-ANCAs of one SSc patient, who had developed a microscopic polyangiitis after aHSCT and therefore received RTX. (B) Course of thrombocytes of one SSc patient, who developed immune thrombocytemia and therefore received RTX. (C) Course of creatine kinase (CK) of a SSc patient, who developed myositis after aHSCT and therefore received RTX; mo, months; MPO-ANCA, myeloperoxidase anti-neutrophilic cytoplasmic antibodies. [file Image_2.tif]
